# Opportunities and Challenges in Advancing Plant Research with Single-cell Omics

**DOI:** 10.1093/gpbjnl/qzae026

**Published:** 2024-03-15

**Authors:** Mohammad Saidur Rhaman, Muhammad Ali, Wenxiu Ye, Bosheng Li

**Affiliations:** Peking University Institute of Advanced Agricultural Sciences, Shandong Laboratory of Advanced Agricultural Sciences in Weifang, Weifang 261325, China; Peking University Institute of Advanced Agricultural Sciences, Shandong Laboratory of Advanced Agricultural Sciences in Weifang, Weifang 261325, China; Peking University Institute of Advanced Agricultural Sciences, Shandong Laboratory of Advanced Agricultural Sciences in Weifang, Weifang 261325, China; Peking University Institute of Advanced Agricultural Sciences, Shandong Laboratory of Advanced Agricultural Sciences in Weifang, Weifang 261325, China

**Keywords:** Multi-omics, Single-cell transcriptomics, Spatial transcriptomics, Single-cell epigenomics, Single-cell proteomics

## Abstract

Plants possess diverse cell types and intricate regulatory mechanisms to adapt to the ever-changing environment of nature. Various strategies have been employed to study cell types and their developmental progressions, including single-cell sequencing methods which provide high-dimensional catalogs to address biological concerns. In recent years, single-cell sequencing technologies in transcriptomics, epigenomics, proteomics, metabolomics, and spatial transcriptomics have been increasingly used in plant science to reveal intricate biological relationships at the single-cell level. However, the application of single-cell technologies to plants is more limited due to the challenges posed by cell structure. This review outlines the advancements in single-cell omics technologies, their implications in plant systems, future research applications, and the challenges of single-cell omics in plant systems.

## Introduction

Heterogeneity in plant cells arises from differentiation during development, as cells acquire distinct characteristics and functionalities. As development progresses, these cells interact and associate with each other, forming complex plant structures [1]. Various omics profiling techniques, such as transcriptomics, genomics, and proteomics, have been employed to study how gene expression varies among different cell and tissue types in bulk samples. However, to gain a deeper understanding of cell differentiation during plant development, single-cell expression profiling has become a popular approach [2–4].

Conventional investigations in biology are conducted from bulk cells, and the outcomes of experiments usually represent the average gene expression of the cell population, or only indicate information about the predominant cell type. Consequently, it is unable to accurately reflect a great deal of information about cell heterogeneity in the sample, disregarding the differences in gene expression regulation between different cell populations as well as between single cells. However, single-cell sequencing, which is sequenced at the distinct cell level, unravels the heterogeneity of genetic diversity between different cells. For instance, various cell types and developmental stages in the *Arabidopsis* roots have recently been identified by single-cell RNA sequencing (scRNA-seq), and the cortex is revealed as the site where brassinosteroids cause a switch from proliferation to elongation, accompanied by increased expression of cell wall-related genes [5]. Additionally, Liu et al. [6] created a cell atlas of soybean nodules and roots by combining single-nucleus transcriptomics and spatial transcriptomics (ST). Using this atlas and the findings of their experiments, they found unusual cell subtypes and their crucial roles in nodule maturation and function. Single-cell techniques have several advantages over bulk techniques, such as the ability to better understand subpopulations of cells and study temporal changes in gene expression in individual cells. However, both methods have their strengths and limitations. In previous plant studies, single-cell techniques have enabled the identification of rare or variable cell populations, discovered specific cell types or states, and provided detailed spatial and temporal gene expression information.

Single-cell omics profiling has emerged as a powerful tool for characterizing cellular heterogeneity in animals and microbes over the past decade [7–9]. Recent advancements in sequencing and protoplast technologies have enabled the study of single-cell transcriptomes in plants [10,11]. These techniques have facilitated the identification of cell types, the creation of expression trajectories, and the elucidation of gene regulatory networks in plants [12–17]. Additionally, single-cell omics approaches have been developed to study various cellular features, including the genome, histone modification, chromatin accessibility, proteome, nucleosome localization, metagenome, and microbiome [18–22].

scRNA-seq has revolutionized transcriptomics by enabling adequate-resolution gene expression analysis of individual cells, facilitating the identification of new cell types and a deeper understanding of cellular heterogeneity [23,24]. However, tissue dissociation is necessary to isolate single cells from their original spatial context, potentially compromising information about their developmental history and spatial relationships. To overcome this limitation, ST was developed as a sequencing technology that can capture both the spatial location and gene expression of individual cells [25]. By combining ST with scRNA-seq, it is now possible to create a comprehensive spatial atlas of gene expression in plants, which has broad implications for the field of plant research. Despite these advances, there is still significant opportunity for growth in single-cell omics research in plants compared with animal studies [26].

In this review, we present a summary of single-cell omics technologies and their uses in plant research. We also discuss the difficulties that single-cell approaches still face and how they will influence and speed up plant-specific research.

## Single-cell transcriptomics technologies and their applications in plants

The scRNA-seq technology has made it possible to characterize every transcript present in a single cell using high-throughput transcriptome sequencing [23]. This technology has the ability to reveal the basic principles of gene regulation, identify various cell types and their roles, and offer new insights into how developmental processes take place in diverse biological samples. However, the effective physical isolation of specific cells remains a significant procedural hurdle for scRNA-seq technology, particularly in plants where a rigid cell wall poses a challenge. Recent advancements in cell wall breakdown by enzymes have enabled the release of protoplasts from plant tissues for single-cell genomic or transcriptomic profiling [27–29]. Alternatively, an intact or cross-linked nucleus can be isolated for profiling [13]. Several cell wall digestion techniques have been developed to liberate protoplasts from various plant tissue types [28,30,31]. However, the enzymatic destruction of the cell wall under hypertonic conditions during protoplast isolation can cause unavoidable disruption to the biological system. Another approach to address these challenges is to optimize the protocol for single-cell preparation, which involves delicate procedures to isolate and extract genetic material from a single cell [32,33]. Other techniques include using specialized sensors and imaging techniques to examine the structure and function of plant cells, which can provide insights into the effects of the cell wall and vacuole on the quality of the genetic material obtained. Overall, addressing these challenges is essential for the successful implementation of single-cell sequencing technologies in plant systems, which has the potential to reveal new insights into the biology and genetics of plant cells.

Recently, single-nucleus RNA sequencing (snRNA-seq) has emerged as a reliable and versatile alternative to protoplast isolation for transcriptomic profiling [13,31,34–36]. The tissues can be preserved or frozen in liquid nitrogen in advance for nucleus isolation, limiting the activation of genes related to stress and other conditions by the isolation process [37]. Despite being more generally applicable and less likely to be impacted by the isolation process, snRNA-seq captures fewer transcripts than scRNA-seq and results in more immature messenger RNAs (mRNAs) [38,39]. In addition, snRNA-seq profiles gene expression in single nuclei rather than in whole cells. In other words, snRNA-seq primarily covers nuclear transcripts, whereas scRNA-seq evaluates cytoplasmic and nuclear transcripts. For the aforementioned reasons, snRNA-seq is more accurate than scRNA-seq in profiling gene expression in cells that are difficult to detach and preserve. Additionally, isolated nuclei provide a comprehensive sampling of cells while avoiding disturbance to the examined biological system. Fresh and preserved tissue and organ samples have been analyzed using snRNA-seq, which has become a powerful tool for determining the role of key regulators in both plants and animals [35,40,41].

In recent plant single-cell studies, the use of nuclei instead of protoplasts is increasing due to their ease of isolation and fast-fixing properties. However, it is notable that protoplasts offer a higher unique molecular identifier (UMI) number than nuclei, resulting in a higher average number of detected genes in cell profiles compared with nucleus profiles. In contrast, nucleus profiling has the ability to capture cells from tissues that are resistant to enzymatic digestion, thereby improving cell identity representation. Recently, a unique cluster was found in nucleus profiling which was not detected in cell profiling [42]. Therefore, protoplasts are better suited for investigating biological questions related to nuclear–cytoplasmic coordination, prolonged progress, and the entire transcriptome set, such as storage RNAs in phase change granules, while nuclei are more useful for examining transient changes and performing snapshot transcriptomic profiling in tissues that are resistant to protoplast isolation. It has been observed that non-negligible *Arabidopsis* leaf protoplast cells can quickly induce the expression of *WOX2* and other genes during protoplast isolation, leading to protoplast regeneration. This suggests that protoplasts may not accurately signify the original transcriptome of the cells [13,43].

**Figure 1:**
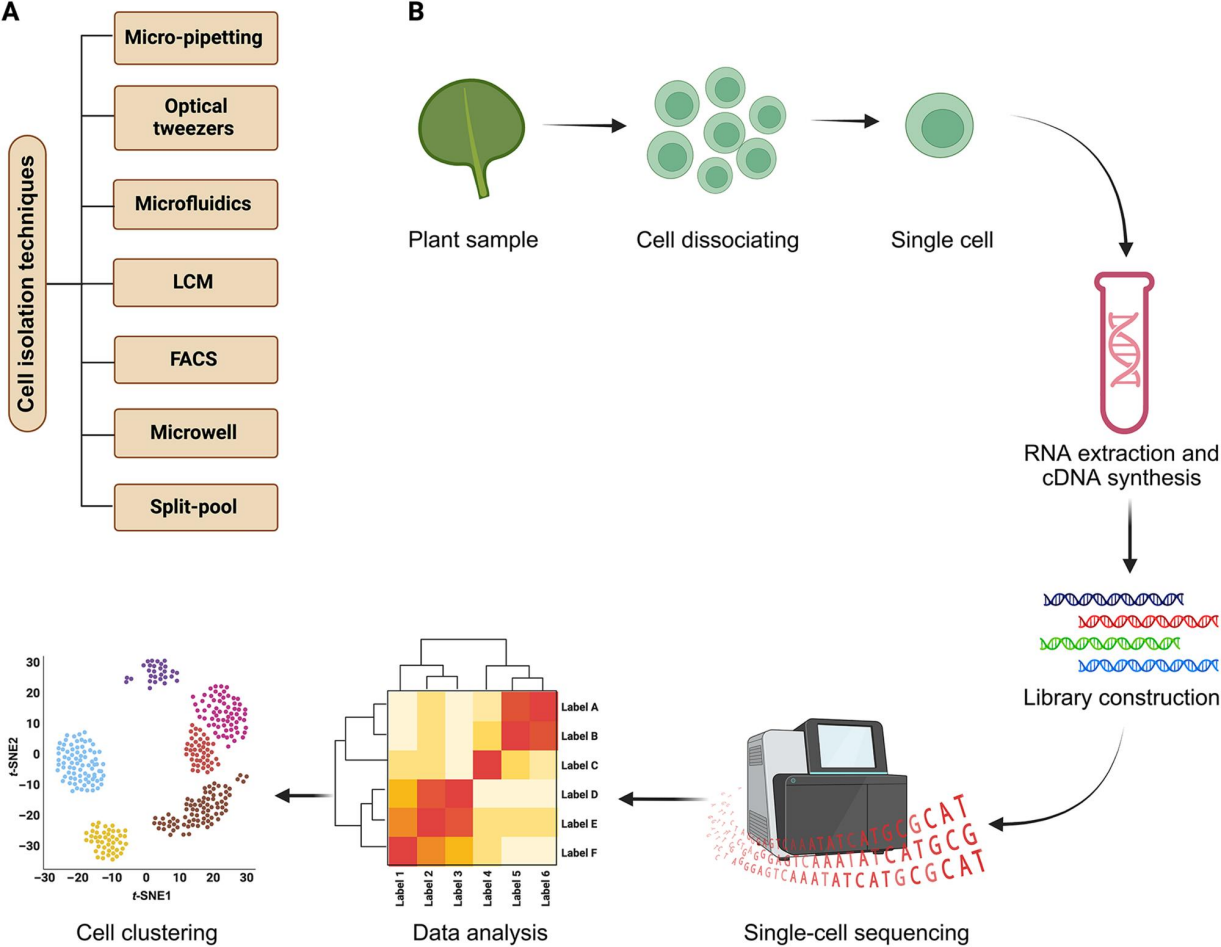
Schematics diagram showing scRNA-seq in leaves **A**. Available single-cell isolation techniques include micro-pipetting, optical tweezers, microfluidics, LCM, FACS, microwell, and split-pool. **B**. Diagram showing scRNA-seq in leaves. The process includes tissue dissection, isolation of single cells, RNA extraction and cDNA synthesis, single-cell sequencing, data analysis, and cell clustering. scRNA-seq, single-cell RNA sequencing; LCM, laser capture microdissection; FACS, fluorescence-activated cell sorting; cDNA, complementary DNA; *t*-SNE, *t*-distributed stochastic neighbour embedding.

Various methods have been developed to isolate individual cells, protoplasts, or nuclei from tissues, cell lysates, or nuclear lysates such as micro-pipetting [44], optical tweezers [45], microfluidics [46], laser capture microdissection (LCM) [47], fluorescence-activated cell sorting (FACS) [48], microwell [49], and split-pool [50] (**Figure 1**A). Traditional techniques such as FACS and LCM have been widely used to separate specific cell types, but the lengthy cell isolation process can cause localized damage to cells with mechanical sorting and laser cutting [51–53]. Furthermore, some fluorescent reporters are challenging to sort using these methods [54]. To address these limitations, microfluidics and droplet-based assays have been developed to isolate single protoplasts or nuclei from a mixed cell suspension [55,56]. Although microfluidics-based isolation technologies offer high-throughput and high-precision capabilities, they require specimens with low levels of contaminants and are more expensive than other techniques. Alternatively, less commonly used techniques such as micro-pipetting, microwells, optical tweezers, and split-pool barcoding are available, and play essential roles in exceptional cases (Figure 1A).

The scRNA-seq has made it possible to analyze the transcriptomes of over 10,000 cells in a single experiment, with the first datasets containing more than one million cells already published [57–59]. This offers an opportunity to conduct high-throughput screens for genetic, signaling, and drug perturbations using hundreds or thousands of samples. However, to study the time-dependent behavior of the heterogeneous cell populations under various perturbations, tens of samples need to be processed simultaneously due to the increasing throughput [60,61]. The current methodologies require parallel handling of numerous individual samples, which is labor-intensive and reagent-intensive. To overcome this limitation, Gehring et al. [62] recently developed an affordable sample multiplexing technique for scRNA-seq, where fixed tissues are chemically tagged with distinguishing DNA oligonucleotides in a one-pot, two-step cross-linking reaction. This approach extends the application of scRNA-seq beyond bulk tissue profiling, enabling the comparison of complex experimental specimens at previously unattainable levels and scales. Additionally, it reduces the constraints imposed by device usage, high chemical cost, and batch effects. However, this approach has only been employed in combination with scRNA-seq/snRNA-seq, and has not yet been applied and may be incompatible with single-nucleus assay for transposase-accessible chromatin with high-throughput sequencing (snATAC-seq). In this regard, Fang et al. [63] proposed a concanavalin A-based sample barcoding (CASB) strategy that can be used jointly with both scRNA-seq/snATAC-seq methods. The CASB approach demonstrates excellent precision in labeling cells or nuclei, independent of background genes, and can resolve cell doublets while simplifying the integration of scRNA-seq/snATAC-seq workflow. In addition, the CASB strategy requires minimal sample preparation, reduces the cost of reagents, improves data analysis performance, and decreases batch effects. All of these advantages preserve the integrity of transcriptomic or epigenomic patterns.

Various methods have been employed in animal cells to understand how genetic variation between individuals affects gene expression in a cell type-specific manner. Studies such as eQTL-Gen, CAGE, and ImmVar have identified expression quantitative trait loci (eQTLs) in diverse cell types and tissues using bulk RNA sequencing (RNA-seq) approaches, where gene expression levels reflect the average signal across many cells [64–66]. While these ensemble analyses provide reliable results, they do not explore gene expression heterogeneity between individual cells. Recently, Yazar et al. [67] used a combination of population genetics and scRNA-seq data to investigate the factors contributing to inter-individual variation in the human immune system. They demonstrated how segregating genetic variation can affect the expression of genes involved in critical immune regulatory and signaling pathways in a cell type-specific manner. As a result, infections, cancer, transplantation, and autoimmune illnesses can all be treated more effectively by understanding the genetic basis of immune system control. However, to our knowledge, there is no report available on plant cells regarding these technologies.

In plant studies, scRNA-seq has been utilized to explore various plant tissues, including leaves, roots, stems, and shoots (Figure 1B; **Table 1**). For instance, Efroni et al. [48], Ryu et al. [68], and Shahan et al. [69] profiled the transcriptomes of individual *Arabidopsis* root cells to construct a single-cell resolution map of the root and investigate the development of root stem cells. Similarly, scRNA-seq was used to analyze the transcriptomes of cells from the stomatal lineage in *Arabidopsis* [14]. Furthermore, Satterlee et al. [11] applied scRNA-seq to study the transcriptomes of maize shoot stem cell progeny and their niche. Additionally, scRNA-seq was utilized to reconstruct the maize meiotic stamen developmental program [70] and to identify the signaling networks that control grass stomata growth and movement [71]. Furthermore, Zhang et al. [72] employed scRNA-seq to survey rice radicals and discovered the mechanisms that regulate cell fate in various cell lineages by profiling distinct root tip cells. Finally, scRNA-seq has also been used to investigate the growth patterns and response mechanisms of other plant species (Table 1) [13,73]. The continued application of scRNA-seq in various plant species shows promising prospects for the future use of single-cell transcriptomics in crop species, which can have implications for applied plant research and contribute to the advancement of our current and future agricultural systems.

**Table 1:** List of scRNA-seq studies in different plant species

Plant species	Cell/tissue type	Refs.
*Arabidopsis thaliana*	Root	[12,35,48,68,69,74–76]
	Shoot apex	[17]
	Leaf	[1,27,77–79]
	Stomata	[80]
*Brassica pekinensis*	Leaf	[41]
*Catharanthus roseus*	Leaf	[81]
*Eucalyptus grandis*	Xylem	[82]
*Glycine max*	Root	[6]
*Gossypium bickii*	Cotyledon	[83]
*Hevea brasiliensis*	Leaf	[84]
*Lotus japonicus*	Root	[85]
*Liriodendron chinense*	Xylem	[86]
*Nicotiana attenuate*	Corolla cell	[73]
*Oryza sativa*	Root and leaf	[28,72,85]
*Pisum sativum*	Shoot	[82]
*Populus trichocarpa*	Xylem	[86]
*Populus* spp.	Stem cell	[87]
*Solanum lycopersicum*	Shoot apex	[13]
*Trochodendron aralioides*	Xylem	[86]
*Zea mays*	Shoot stem	[11]
	Stomata	[71]
	Root	[36,88,89]

*Note*: scRNA-seq, single-cell RNA sequencing.

## Single-cell epigenomics technologies and their applications in plants

Single-cell epigenomics investigates epigenetic modifications in single cells rather than in bulk cells, which allows an in-depth understanding of the cellular heterogeneity and the epigenetic mechanisms contributing to the regulation of gene expression in various organisms. The basic principle of single-cell epigenomics includes cell isolation, genome amplification, epigenetic profiling (such as DNA methylation or chromatin accessibility), and sequencing analysis (involving data normalization, dimensionality reduction, clustering, and visualization of epigenetic profiles) [90]. The integration of single-cell epigenomic data with other data types (*e.g.*, transcriptomic data) can provide a multi-omic view of cellular function [91]. It involves the study of chromatin accessibility, three-dimensional (3D) genomic architecture, DNA methylation, histone modifications, and DNA–protein interactions at the genomic level. Single-cell epigenome sequencing technologies have advanced rapidly, offering a more comprehensive understanding of cell type-specific gene regulation mechanisms. For instance, while 5-methylcytosine (5mC) at CpG dinucleotide has been previously used as an epigenetic marker, it has context-dependent influences on transcription in addition to transcriptional inhibition. Recently, bisulfite-converted randomly integrated fragments sequencing (BRIF-seq) has become widely used for detecting DNA methylation, which can distinguish between methylated and unmethylated cytosines in genomic DNA. Compared with conventional single-cell bisulfite sequencing (scBS-seq), BRIF-seq produces more evenly dispersed reads across the genome, particularly in highly methylated and repetitive regions such as maize microspores. This facilitates the identification of heterogeneous locations and enables high rates of read mapping and genome coverage [92].

Chromatin accessibility refers to the ability of DNA to be accessed by regulatory factors, which can be detected by two sequencing technologies: deoxyribonuclease I hypersensitive site sequencing (DNase-seq) and assay for transposase-accessible chromatin with high-throughput sequencing (ATAC-seq) [93,94]. Single-cell ATAC-seq (scATAC-seq) utilizes the ATAC-seq of sorted single nuclei to explore open chromatin regions at the single-cell level [93]. By integrating scATAC-seq with combinative indexing of undamaged nuclei, chromatin accessibility can be quantified in single cells [95]. For example, scATAC-seq has been used to determine chromatin accessibility in the nuclei of maize organs [36]. Dorrity et al. [15] identified three distinct developmental phases of endodermal cells and constructed single-cell maps of open chromatins in *Arabidopsis* roots by integrating scATAC-seq data with scRNA-seq data. Moreover, a microfluidics-based 10X Genomics Chromium scATAC system can categorize single nuclei by binding them with barcoded gel beads [96]. Recently, high-resolution nucleosome occupancy and methylome sequencing (NOMe-seq), single-cell chromatin overall omic-scale landscape sequencing (scCOOL-seq), and single-cell nucleosome, methylation, and transcription sequencing (scNMT-seq) have been developed to identify chromatin state and DNA methylation simultaneously [97–99]. However, the use of these modern sequencing tools in plant research is limited. Incorporating these technologies in plant studies will provide valuable insights into chromatin accessibility at the single-cell level, promoting the advancement of functional genomics research.

Chromatin immunoprecipitation coupled with high-throughput sequencing (ChIP-seq) and cleavage under targets and tagmentation (CUT&Tag) methods are frequently used to locate genome-wide histone alterations [100,101]. Furthermore, ChIP-seq is utilized to examine additional protein–DNA interactions and transcription factor binding sites [102]. Single-cell epigenomics has been successfully applied in animals following the development of techniques like single-cell CUT&Tag-pro [103], CoBATCH [104], and 10X scCUT&Tag [105,106]. However, it is challenging to get single cells in plants because of the presence of cell walls. The existing chromatin immunocleavage (ChIC) approaches, on the other hand, necessitate numerous single-cell barcoding procedures following tagmentation, which may result in some DNA leakage and decrease the mapping effectiveness. Therefore, a reliable single-cell ChIC sequencing approach is needed for plant epigenomics research. The snCUT&Tag assay and scATAC were recently coupled to provide a quick ChIC-based chromatin profiling methodology, which led to the development of the simple-to-use single-nucleus CUT&Tag (snCUT&Tag) approach in rice. The use of these aforementioned methods in different plant species studies will thus be a developing field.

In addition, single-cell combinatorial indexed high-throughput chromosomal conformation capture (Hi-C) has been used to define 3D genomic architecture [107]. It has been reported that isolated single-cell chromatin conformation has been depicted and recreated using Hi-C [108]. Recent research has enhanced single-cell Hi-C resolution and provided solid proof of heterogeneity within cells [109]. This method labels DNA in intact nuclei and enables high-throughput studies using nucleic acid barcodes from subsequent rounds [107]. During fertilization, chromatin conformation reconfiguration in rice eggs, sperm cells, unicellular zygotes, and shoot mesophyll cells has been determined through research into single-cell 3D genomes [110].

Consequently, when single-cell epigenome sequencing tools evolve further, we will be able to understand many plant developmental processes at the single-cell level. Yet, further studies on single cells in various plant species are warranted.

## Single-cell proteomics technologies and their applications in plants

Single-cell proteomics is an emerging field that aims to identify and quantify the proteomes of individual cells. The principle behind these technologies involves the isolation of individual cells from a population, followed by the analysis of their protein contents using various mass spectrometry (MS)-based methods [111]. Single-cell proteomics technologies are particularly useful in elucidating the biological heterogeneity and protein expression patterns of individual cells, with potential applications in cancer research, regenerative medicine, and microbial ecology, among other fields. The analysis process of single-cell proteomics involves data processing and analysis to identify and quantify proteins of interest by comparing protein expression levels across individual cells or cell populations, integrating proteomic data with other omics data, and applying machine learning algorithms to identify biomarkers and patterns of disease. Unfortunately, proteomics studies at the single-cell level in plants have been limited due to the technical challenges of isolating sufficient numbers and types of cells from plant tissues. Previous investigations have examined the single-cell proteomes using mature or germinating pollen grains from *Arabidopsis*, rice, maize, and tomato [112–115], providing valuable insights into the molecular mechanisms driving pollen development, germination, pollen tube growth, and pollen–stigma interaction. Isotope-coded affinity tags and isobaric tags which are used for relative and absolute quantitation have also been used for quantitative analysis in pollen and guard cell research [116,117]. In the root tip of a tomato under aluminum (Al) stress, LCM and tandem mass tag (TMT) cell-specific proteomic analyses were performed to identify numerous proteins and their dynamic changes directly related to Al resistance [118]. Although proteomics studies at the single-cell level in plants remain limited, these studies demonstrate the potential for proteomic analysis to further our understanding of plant development and physiology at the single-cell level.

Recent technological advancements have led to significant progress in proteomics research, particularly in animal cells. For instance, combining nano-droplet sample preparation, high-field asymmetric waveform ion mobility spectrometry (FAIMS), and the latest Orbitrap Eclipse Tribrid mass spectrometer techniques enabled the identification of up to 1000 protein groups in individual HeLa and motor neuron cells [119]. Additionally, the recent development of single-cell proteomics by MS (SCoPE2-MS) has allowed for the measurement of approximately 3000 proteins in over 1500 cells, representing one of the most comprehensive single-cell human proteomes to date [120]. Therefore, applying these cutting-edge proteomics methods to study plant cells to unravel the dynamic alterations of proteins under stressful and non-stressful conditions is crucial.

## Single-cell metabolomics technologies and their applications in plants

The metabolome encompasses all low-molecular-weight metabolites produced by a cell and has the potential to serve as an important indicator of cell state by revealing the cell’s specific metabolic processes and conditions. Despite its function, measuring the metabolome at the single-cell level presents significant challenges owing to the highly dynamic nature of the system and the limited availability of techniques for accurate detection, tagging, and amplification of small molecules [121]. Recent advancements in optical nanosensors, expression systems, and *in vivo* imaging technology have facilitated real-time measurement of metabolite concentrations at the single-cell level [122]. Furthermore, advancements in single-cell mass spectrometers have expanded the scope of metabolomics research to analyze metabolites on a cellular and subcellular level. For instance, the application of single-cell capillary electrophoresis coupled with electrospray ionization time-of-flight MS has enabled the quantification of metabolites in individual isolated neurons. Additionally, researchers have applied the microarrays for MS (MAMS) platform to validate single-cell metabolite analysis and monitor cellular responses to genetic and environmental stressors [123]. By understanding the single-cell metabolome, researchers can gain a better understanding of cellular metabolism as well as its impact on cellular behavior and its role in different plants.

Plant metabolomics studies have highlighted the significance of cell-specific metabolism in controlling vital physiological developments in plants, such as the development of specialized metabolism, C4 metabolism, regulation of stomatal closure, and shoot apical meristem metabolism [104,124–126]. However, such studies often use labor-intensive protocols to isolate specific cells or reporter lines to target a few specific metabolites, making true metabolomics at the cellular level challenging due to the large number of metabolites involved. Furthermore, gas chromatography MS is widely used in metabolomics due to its elevated resolution, excellent specificity, wide-ranging application, and high sensitivity [127]. During the process of extraction, purification, and enrichment, the spatial dissemination of metabolites in tissues can vanish, which limits its usefulness. Molecular imaging using mass spectrometry imaging (MSI) is a novel tactic that provides tidings on the content, configuration, and spatial distribution of both known and undiscovered endogenous metabolites in biological tissues, generating tissue molecular imaging maps [128].

With the passage of time and the advancement of technology, various imaging techniques are widely used in plants. An adhesive film has been implemented to effectively improve desorption electrospray ionization MSI (DESI-MSI) analysis in the analysis of strawberry amino acids, sugars, organic acids, and specialized metabolites such as anthocyanins and flavan-3-ols [129]. Liu et al. [28] demonstrated that a reliable and suitable sample preparation tactic is essential for subsequent MS analysis, by utilizing caffeic acid as a standard organic acid matrix for endogenous protein imaging of soybean seeds using marix-assisted laser desorption ionization MSI (MALDI-MSI). Sarabia et al. [130] employed MALDI-MSI, liquid chromatography MS, and inductively coupled plasma MS and investigated metabolites and lipid distribution in the roots of barley seedlings under salinity stress. Overall, MSI is a powerful technique for metabolomics research in plants, with a wide range of applications and potential advances.

Although it has some benefits regarding *in situ* analysis, the MSI platforms provide substantial challenges, particularly in data analysis [131]. Despite being a relatively new technique, MSI lacks data processing standards like normalization. Additionally, it was only recently that an open cross-platform data format was introduced [132], complicating the process of quantification. Publications that report absolute metabolite levels using MSI are scarce, exemplified by the work of Shroff and his colleagues [133].

## ST and its applications in plants

**Figure 2:**
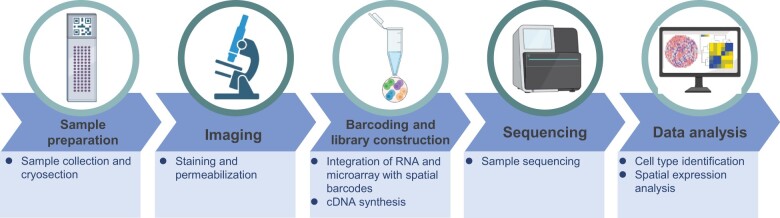
Workflow of 10X Visium ST analysis ST, spatial transcriptomics.

ST aims to integrate spatial and transcriptomic information to identify the cellular architecture of a tissue. The principles of ST include the capture of spatial information and transcriptomic data and the integration of both datasets, whereas the experimental process involves several steps, including tissue preparation, tissue capture, spatial barcoding, RNA isolation, and sequencing analysis. The optimization of each step is critical to achieving high-resolution data and detecting rare cell populations. The data analytical process of ST comprises computational processing and statistical analysis to identify the localization of transcripts in the tissue through data normalization, segmentation, clustering, and visualization of spatial transcriptomic profiles. The emergence of ST has allowed for preserving of spatial information during sequencing [25,134]. Advances in technology have made it possible to visualize gene expression in cells within their tissue context with high resolution, including *in situ* hybridization (ISH), single-molecule RNA fluorescent ISH, and LCM [135–137], although these techniques have limitations and are time-consuming. New technologies such as 10X Visium, BGI spatial enhanced resolution omics sequencing (Stereo-seq), and Slide-seq have made ST more accessible (**Figure 2**) [138]. One challenge in ST is the preservation of tissue morphology, and researchers have used methods such as cryosectioning with a thin coating of Tissue-Tek optimal cutting temperature compound to manage tissue structure [139,140]. Spatial barcodes are used in 10X Visium ST, Slide-seq, and high-definition ST to collect RNA before sequencing and provide next generation sequencing (NGS)-encoded location data regarding transcripts, which can be used to produce spatial information for the entire transcriptome in an unbiased manner [141,142]. These advancements in ST will enable the visualization of gene expression in their spatial context, providing new insights into cellular functions and interactions within tissues.

**Figure 3:**
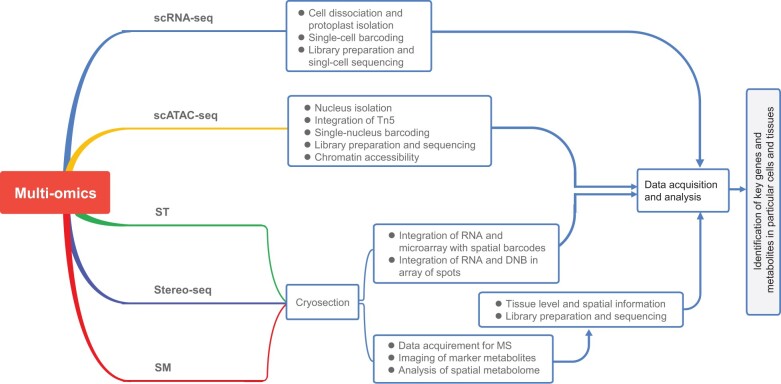
A proposed flowchart for multi-omics study in plants scATAC-seq, single-cell assay for transposase-accessible chromatin with high-throughput sequencing; Stereo-seq, spatial enhanced resolution omics sequencing; SM, spatial metabolomics; DNB, DNA nanoball.

Although ST technologies have been developed to overcome the lack of spatial information in conventional scRNA-seq, their applications in plants have been limited to few studies (**Table 2**), likely due to the challenges with plant cell wall breakdown and transcript diffusion to the array surface [143]. Additionally, the single-cell resolution has not been achieved with 10X Visium ST [144]. Nonetheless, ST techniques have successfully created transcriptome-wide spatial gene expression profiles in several plants, including *Arabidopsis*, *Populus tremula*, and *Picea abies* [5,139,143,145]. Several recent studies have utilized various spatial omics technologies to gain insights into plant biology. Liu et al. [14] utilized 10X Visium technology to investigate the gene activity and molecular architecture of flower organogenesis in orchids. Nolan et al. [5] identified the gene regulatory networks involved in brassinosteroid response in *Arabidopsis* roots using 10X Genomics Chromium technology, revealing a spatial response to brassinosteroids in the cortex. Peirats-Llobet et al. [146] used 10X Visium technology to study the spatiotemporal regulation of gene expression in different seed cell types of growing barley grains, while Moreno-Villena et al. [147] used LCM and 10X Visium to analyze spatial gene expression patterns in *Portulaca oleracea* leaves under water deficit circumstances to investigate the C4 and crassulacean acid metabolism (CAM) photosynthesis system in plants. Song et al. [148] employed BGI Stereo-seq, BMKMANU S1000, and 10X Visium technologies to study the spatial transcriptome of tomato callus during shoot regeneration, providing insights into cell type differentiation and vascular tissue development. Single-cell Stereo-seq (scStereo-seq) has also been used to distinguish between highly similar cell subtypes and identify spatial gradients in the expression of genes relevant to photosynthesis in *Arabidopsis* [77]. However, more research is necessary to fully explore the potential of these spatial omics tools in plant biology. Nevertheless, further research can be conducted using a combination of recently developed spatial omics tools. As a consequence, we suggested a potential multi-omics analysis workflow that would include several techniques, including scRNA-seq, 10X Visium ST, Stereo-seq, and air flow-assisted ionization imaging MS-based spatial metabolomics (**Figure 3**).

**Table 2:** List of ST studies in different plant species

Plant species	Cell/tissue type	Sequencing technique	Ref.
*Arabidopsis thaliana*	Root	10X Visium	[5]
	Inflorescence meristem	10X Genomics	[143]
	Epidermal cell	scStereo-seq	[77]
	Leaf	MERFISH	[78]
*Glycine max*	Root	Stereo-seq	[6]
*Hordeum vulgare*	Seed	10X Visium	[146]
*Medicago truncatula*	Nodule	10X Visium	[66]
*Phalaenopsis Big* Chili.	Floral organ	10X Visium	[1]
*Picea abies*	Female cone	10X Genomics	[143]
*Populus* spp.	Stem	10X Visium	[145]
*Populus tremula*	Leaf bud	10X Genomics	[143]
*Portulaca oleracea*	Leaf	LCM and 10X Visium	[147]
*Solanum lycopersicum*	Callus (shoot)	BGI Stereo-seq, BMKMANU S1000, and 10X Visium	[148]

*Note*: ST, spatial transcriptomics; Stereo-seq, spatial enhanced resolution omics sequencing; scStereo-seq, single-cell spatial enhanced resolution omics sequencing; LCM, laser capture microdissection; MERFISH, multiplexed error-robust fluorescence *in situ* hybridization.

## Analytical approaches for processing single-cell omics data

The basic principle of single-cell technologies is to label cell-specific barcodes and transcript-specific barcodes (*i.e.*, UMIs) for each cell. Enzymatic cell wall digestion and artificial separation are the two main methods for obtaining single cells or single nuclei from plant tissues. Separate single cells or single nuclei are labeled with unique labels using different techniques, such as 10X Genomics, Smart-seq, and split-pool ligation-based transcriptome sequencing (SPLiT-seq) [12,50,80]. The labeled transcript is constructed into a library and subsequently sequenced. For single-cell genomics and ST, sequencing saturation needs to be predicted based on sequencing depth and sample complexity. Generally, raw reads should exceed 100 billion nucleotides. The target can detect 500–10,000 cells, and the use of UMIs and median gene numbers also depends on the research question and sample, without a unified standard. scRNA-seq ultimately generates a complex and high-dimensional gene expression matrix. Various tools have been developed for generating the gene expression matrix, such as Seurat [149] and Monocle [59] based on R and SCANPY [150] using Python. To improve the signal-to-noise ratio, the matrix requires pre-processing, including filtering out low-quality cells, normalization and scaling of data, and detecting highly variable features. For data dimension reduction and visualization, the scaled data are usually subject to principal component analysis (PCA) linear dimensionality reduction, *t*-distributed stochastic neighbour embedding (*t*-SNE), and uniform manifold approximation and projection (UMAP) non-linear dimensionality reduction. These results are then used for cell clustering using techniques like modularity optimization [*e.g.*, the *k*-nearest neighbor (KNN) algorithm, Louvain algorithm, or smart local moving] and subsequent cell type annotation based on differentially expressed genes. Currently, multiple plant cell marker databases exist to assist with cell type annotation [151,152].

Cell clustering is used to discover new cell types and characterize their gene expression patterns. For example, procambium-like cells in poplar stem, the newly defined cell type, are located within the phloem domain and differentiate into phloem cells [145]. In addition, the captured cell population theoretically contains many or continuous differentiation states. The cell development trajectory is obtained by sorting the cell expression profile in pseudotime using tools such as Monocle. RNA velocity, which adds splicing information, is a method used to infer the direction of cell differentiation, such as scVelo [153] and Dynamo [154]. For example, the differentiation trajectory of uninfected cells in soybean root nodules was revealed by combining pseudotime and RNA velocity analysis [6].

The scRNA-seq defines cell type according to marker gene(s), but cannot capture its spatial information. At present, the mainstream spatial RNA sequencing (spRNA-seq) uses spatial barcodes to replace cell-specific barcodes in scRNA-seq, such as 10X Genomics and Stereo-seq [145,155]. High-resolution ST technology enables *in situ* single-cell transcriptomic profiling, such as Stereo-seq [155]. The scRNA-seq analysis method is completely applicable to the spRNA-seq analysis. In addition, spatial information is also applied to cell clustering, and it contributes to precise classification of cell types. For example, upper and lower epidermal cell types in *Arabidopsis* leaves were distinguished by combining single-cell methods and spatial information [77]. The spatial information provided by spRNA-seq enables it to conduct a wider range of biological analyses compared with scRNA-seq/snRNA-seq, including intercellular communication, co-localization of gene expression regions, and characterization of gene expression in specific tissues. While relevant reviews provide a good summary of spRNA-seq analysis, there is a need for the development of analysis software specifically tailored for spRNA-seq and distinct from scRNA-seq [156,157].

Network analysis displaying gene regulatory networks is commonly used in bulk transcriptome analysis, and similar tools are available for single-cell transcriptomes. The hdWGCNA is a method using KNN algorithms for detecting and investigating highly correlated gene clusters based upon high-throughput sequencing expression datasets, and performs weighted gene co-expression network analysis (WGCNA) after reducing the sparsity of the data [106,158,159]. In addition to conventional gene interaction networks, hdWGCNA can be visualized using UMAP for display of the gene network diagram [160]. Based on the power of network analysis, it can be utilized for integrating and analyzing multiple single-cell omics data.

## Limitations of omics technologies in plant systems and future research applications

The interest of plant researchers is increasing in single-cell omics techniques, but several obstacles still need to be overcome to achieve their full potential. Although established approaches from animal models have simplified the initial application, adapting them for plant cells requires additional care and effort. Sample preparation and cell isolation are critical challenges when implementing single-cell tactics in plant research, as different species and tissues may require different preparation strategies. Although enzyme combinations have been used to remove cell walls in some species and tissues [28,30,31], the composition of cell walls varies, and enzymatic digestion is not always practical. These procedures can also be time-consuming since cell walls must be gradually broken down using enzymes to protect the protoplasts. Protoplasts must be processed quickly after isolation, because they are sensitive to mechanical and environmental stresses, which can interfere with subsequent tests. Therefore, it is necessary to develop better cell separation techniques to enable the widespread use of single-cell omics and to prevent later contamination [43].

Single-cell omics poses another challenge in detecting and identifying cell type-specific marker genes. To be effective, these markers must be expressed continuously in the target cell type, which can be challenging due to the stochasticity and noise in expression measurements compared with RNA-seq data [161]. Moreover, a curated collection of marker genes specific to the organisms and cell types of interest is also necessary. Identifying cell type-specific marker genes in plants can be challenging, as many plants lack them, although some cell types in *Arabidopsis* roots have been found to have a range of markers. Most plant cell type-specific profiling has been done at the transcript level [162], and other omics datasets, such as proteomics, currently have fewer markers. Combining these single-cell proteomics approaches with cell type-specific profiling, as they are developed and applied in plant systems, could help identify the required markers for single-cell cataloging.

Another issue with scRNA-seq is the loss of spatial information of cells within a tissue during the cell isolation process [163,164]. To overcome this challenge, researchers have employed several early techniques such as ISH, single-molecule RNA fluorescence ISH, and LCM to visualize gene expression in cells within their tissue context [36]. More recently, technologies like 10X Visium and Slide-seq have emerged, facilitating the use of ST [138], which is a novel technology with the potential to provide spatial information and minimize the loss of cell types that can occur during single-cell analysis [138]. ST and imaging of tissue sections can enable the investigation of cell–cell interactions within tissues and the spatial distribution of gene expression [143]. However, single-cell and spatial biology is relatively new fields in plant research, and technical limitations may affect their results. One of these limitations is cryoinjury caused by the crystallization and recrystallization of water molecules in cells, which has been largely overlooked in single-cell and spatial biology. Cryo-electron tomography (cryo-ET) is starting to address this issue by studying protein structures *in situ*, requiring intact cells at the nanometer scale [165]. Although single-cell and spatial biology typically operates on a scale of 0.5–100 µm, the Leidenfrost effect and slow freezing of optimal cutting temperature (OCT) embedment can lead to reversible and uneven damage to cells or nuclei. To avoid this, using fresh materials and isolating cells/nuclei at room temperature are recommended. Cryoinjury limits the ability of array-based ST to achieve single-cell and subcellular resolutions, which can be overcome using high-pressure freeze (HPF) technology that rapidly freezes water in cells to form “vitreous ice”. Future plant research may use formalin-fixed paraffin-embedded (FFPE) tissue specimens coupled with advanced spatial technology. However, plant spatial biology faces challenges in transferring tissue sections to spatial slides, cell border recognition, multi-layer image scanning, and precise matching. Furthermore, both single-cell transcriptomics and ST can detect a small number of genes and low UMIs, and even do not detect subcellular resolution. However, some potential solutions include improving capturing efficiency and utilizing the latest technologies such as 10X Xenium for subcellular resolution. This technique requires higher resolution, sensitivity, and validation of gene expression in particular cell types. Although ISH and fluorescence-based reporting lines essentially meet these parameters, there is always room for improvement in these technologies.

## Concluding remarks

**Figure 4:**
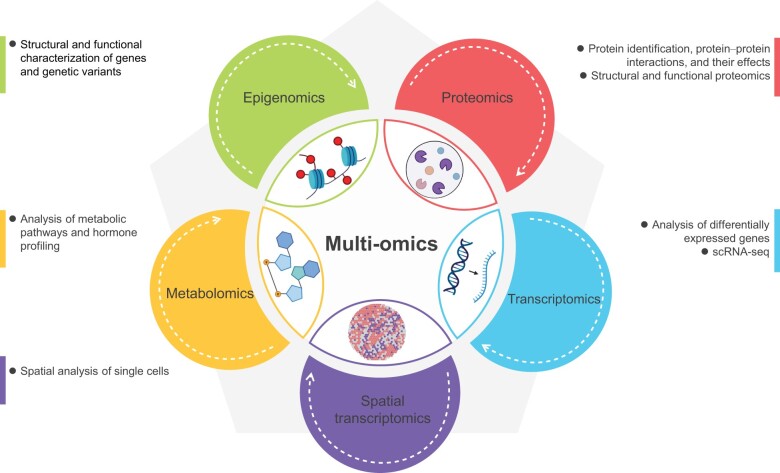
The use of multi-omics techniques to study gene expression, protein abundance, metabolite levels, and epigenetic modifications in plants

Single-cell omics technologies and ST have revolutionized the study of plant cells by providing an in-depth analysis of the molecular and functional heterogeneity of individual cells in complex multicellular tissues. Single-cell omics allows the analysis of individual plant cells, providing a more precise view of cell heterogeneity and the underlying molecular mechanisms involved in plant development, growth, and adaptation to stress. ST, on the other hand, enables the direct visualization of the transcriptomes of hundreds of cells in their native context, contributing to the understanding of cell–cell interactions, cell–cell communication, and non-cell-autonomous processes. These technologies have a wide range of applications, including the discovery of rare cell types, the identification of molecular markers for plant breeding, the discovery of stress-responsive genes and regulatory networks, and the identification of potential targets for crop improvement (**Figure 4**). Furthermore, these technologies provide a comprehensive understanding of the spatial distribution of gene expression, providing a deeper understanding of the molecular basis of tissue-specific and organ-specific functions. Hence, integration of single-cell omics and ST can lead to unprecedented insights into plant cell biology, thereby improving crop yields, and addressing critical issues related to sustainable agriculture.

## CRediT author statement

**Mohammad Saidur Rhaman:** Conceptualization, Investigation, Writing – original draft, Writing – review & editing. **Muhammad Ali:** Conceptualization, Investigation, Writing – original draft, Writing – review & editing. **Wenxiu Ye:** Supervision, Conceptualization. **Bosheng Li:** Supervision, Conceptualization, Writing – review & editing. All authors have read and approved the final manuscript.

## Competing interests

The authors have declared no competing interests.
